# Pediatric Hepatoblastoma: From Developmental Molecular Mechanisms to Innovative Therapeutic Strategies

**DOI:** 10.3390/cancers18050879

**Published:** 2026-03-09

**Authors:** Ana Maria Scurtu, Elena Țarcă, Laura Mihaela Trandafir, Alina Belu, Alina Jehac, Ioana Martu, Valentin Bernic, Rodica Elena Heredea, Viorel Țarcă, Dumitrel Băiceanu, Elena Cojocaru

**Affiliations:** 1 Grigore T. Popa University of Medicine and Pharmacy, 700115 Iasi, Romania; scurtu_ana-maria@d.umfiasi.ro (A.M.S.); laura.trandafir@umfiasi.ro (L.M.T.); belu_alina@d.umfiasi.ro (A.B.); alina.jehac@umfiasi.ro (A.J.); ioana.martu@umfiasi.ro (I.M.); bernic.valentin@d.umfiasi.ro (V.B.); dumib24@gmail.com (D.B.); elena2.cojocaru@umfiasi.ro (E.C.); 2 Department of Clinical Practical Skills, Victor Babeş University of Medicine and Pharmacy, 300041 Timisoara, Romania; elena-rodica.heredea@umft.ro; 3 Faculty of Medicine, Apollonia University, 700511 Iași, Romania; viorel.tarca@univapollonia.ro

**Keywords:** pediatric hepatoblastoma, developmental liver cancer, Wnt/β-catenin signaling, tumor stemness, precision medicine

## Abstract

Background/Objectives: Hepatoblastoma, the most common pediatric primary liver cancer, is no longer regarded as a conventional malignancy but rather as a tumor emerging from disrupted hepatic developmental processes. Although improvements in chemotherapy, surgical techniques, and liver transplantation have markedly enhanced survival, therapeutic decision-making is still primarily guided by anatomical criteria and insufficiently reflects the biological heterogeneity that contributes to variable treatment response and disease recurrence. This narrative review integrates recent advances in molecular biology, tumor stemness, microenvironmental interactions, and translational research models in pediatric hepatoblastoma. We critically examine how developmental signaling pathways, cellular plasticity, and immune–vascular context shape tumor behavior and therapeutic vulnerability, with a focus on emerging targeted, anti-angiogenic, immune, and epigenetic strategies. Results: Hepatoblastoma is characterized by aberrant activation of key developmental pathways, including Wnt/β-catenin, Hippo–YAP, IGF, and mTOR signaling, which cooperate to sustain proliferation, stem-like phenotypes, and treatment resistance. Tumor heterogeneity is further reinforced by cancer stem cell populations and a predominantly immune-cold microenvironment. While innovative therapeutic approaches show promise, their clinical impact has been limited by biological complexity and insufficient integration into current treatment algorithms. Liquid biopsy biomarkers, advanced translational models, and multi-omics approaches offer new opportunities for biologically informed risk stratification and therapy adaptation. Conclusions: Future progress in pediatric hepatoblastoma will require a paradigm shift from purely clinicopathological management toward an integrated molecular and surgical framework. Incorporating biological stratification into therapeutic decision-making may enable personalized treatment, rational therapy de-escalation, and improved outcomes for high-risk disease. This review highlights the foundations and future directions for precision medicine in hepatoblastoma.

## Simple Summary

Pediatric hepatoblastoma is a developmentally driven liver malignancy characterized by aberrant activation of fetal signaling pathways and persistence of stem-like tumor cell populations. Tumor heterogeneity is reinforced by hypoxia, angiogenesis, and an immune-cold microenvironment, contributing to therapeutic resistance and relapse. Integrating molecular biology with clinical and surgical decision-making may enable personalized treatment strategies, rational therapy de-escalation, and improved long-term outcomes. Despite advances in multimodal therapy, current risk stratification still relies predominantly on clinical and imaging-based parameters, with limited incorporation of biological data. A better understanding of tumor biology and its integration into routine practice may help refine patient selection, optimize treatment intensity, and reduce long-term toxicity in survivors.

## 1. Introduction

Hepatoblastoma (HB) represents the most frequent primary malignant tumor of the liver in the pediatric population, with a marked predilection for infants and children younger than five years [1,2]. Recent global burden studies show that HB incidence has increased continuously over recent decades, with substantial variation between geographic regions, and predictions based on current trends suggest that this rising burden is likely to persist through the next decade and beyond [3].

Unlike adult hepatocellular malignancies, HB is widely regarded as an embryonal tumor, arising from disrupted hepatic development rather than from cumulative environmental or inflammatory insults [4]. This developmental origin is reflected in its close association with prematurity, very low birth weight, and congenital syndromes such as Beckwith–Wiedemann syndrome and familial adenomatous polyposis [5]. From a biological perspective, HB recapitulates early stages of liver organogenesis, characterized by aberrant activation of fetal signaling pathways and persistence of immature hepatoblast-like cells [4].

Although survival outcomes have improved markedly over recent decades, primarily due to cisplatin-based chemotherapy, refined surgical approaches, and the availability of liver transplantation, current HB management remains largely guided by clinical considerations [6]. Risk stratification relies mainly on imaging-based systems such as Pretreatment Extent of Disease (PRETEXT), serum alpha-fetoprotein (AFP) levels, and resectability criteria, while therapeutic decisions are guided by tumor burden and anatomical considerations [5]. Although this approach has proven effective in improving overall survival, it fails to fully account for the marked biological heterogeneity observed among HBs. Consequently, a subset of patients experiences chemoresistance, treatment-related toxicity, or disease relapse, underscoring the limitations of a strictly clinicopathological framework [2].

In parallel with clinical advances, molecular studies over the last two decades have revealed HB as a genetically and epigenetically distinct entity, driven by alterations in key developmental pathways, most notably Wnt/β-catenin, Hippo–YAP, IGF, and mTOR signaling [2]. These pathways play fundamental roles in normal liver development and growth control, and their dysregulation contributes to tumor initiation, maintenance of stem-like features, and therapeutic resistance. However, the translation of molecular insights into clinically actionable strategies has remained limited, and molecular profiling is not yet routinely integrated into treatment algorithms or surgical decision-making [7,8].

This gap between molecular understanding and therapeutic implementation represents a significant barrier in clinical management of pediatric HB. While targeted therapies, anti-angiogenic agents, immunotherapy, and epigenetic modulators are increasingly explored in preclinical and early clinical settings, their optimal application requires a biologically informed framework that links molecular mechanisms to tumor behavior and treatment response [9,10]. Bridging this gap is particularly relevant in an era that aims to balance cure rates with long-term quality of life in pediatric cancer survivors.

This review was designed as a narrative synthesis aimed to integrate developmental biology, tumor heterogeneity, and microenvironmental features of HB with current clinical management strategies. A structured literature search was performed using PubMed/MEDLINE, Scopus, and Web of Science databases, focusing on studies published between 2015 and 2025, with inclusion of seminal earlier references where relevant. Search terms included combinations of “hepatoblastoma”, “Wnt/β-catenin”, “Hippo/YAP”, “tumor microenvironment”, “liquid biopsy”, “risk stratification”, and “pediatric liver tumors”.

Given the rarity of HB and the limited number of prospective clinical trials, evidence was prioritized hierarchically, favoring prospective and multicenter clinical studies where available, followed by large retrospective cohorts, translational omics analyses, and pathophysiology-based preclinical models. Attention was given to studies providing clinically actionable insights or biomarker-driven hypotheses. We acknowledge that the integration of heterogeneous evidence represents a limitation, and we clearly distinguish throughout the manuscript between evidence-based conclusions and hypothesis-generating concepts.

## 2. Epidemiology and Clinical Background

Recent epidemiological analyses confirm that incidence of HB remains highest during the first three years of life, with a marked decline subsequently, supporting the concept that tumor initiation is closely linked to early developmental events [11]. A consistent male predominance has been reported across recent cohorts, suggesting that sex-related biological factors may modulate susceptibility during hepatic development [11]. Current population-based studies underscore that the global burden of HB is not uniformly distributed. Incidence and outcomes vary substantially by region, reflecting differences in perinatal risk profiles, diagnostic access, and availability of specialized multidisciplinary care. Recent Global Burden of Disease (GBD 2021)–based work has highlighted marked regional heterogeneity and has suggested overall long-term declines in incidence and mortality from 1990 to 2021 at the global level, while continuing to identify regions where the burden remains disproportionately high [3,12].

Over the last five years, population-based studies have refined the epidemiological profile of HB by emphasizing the role of perinatal and developmental risk factors. Prematurity and very low birth weight have emerged as the strongest non-genetic associations, with affected children displaying a several-fold increased risk compared with those born at term [13,14]. These observations reinforce the hypothesis that HB arises in the context of disturbed liver maturation and regenerative stress during a critical postnatal window, rather than because of long-term environmental exposure. In addition, a subset of cases continues to be linked to hereditary cancer predisposition syndromes, most notably Beckwith–Wiedemann spectrum and familial adenomatous polyposis, underscoring the need for vigilance regarding syndromic features and family history at diagnosis [14,15,16].

Clinically, most children present with a progressive, often painless abdominal mass or distension, sometimes accompanied by nonspecific symptoms such as reduced appetite, weight loss, or irritability; overt jaundice is uncommon and should prompt careful consideration of differential diagnoses and biliary involvement [17]. At presentation, many HBs cannot be safely resected upfront due to extensive tumor burden, multifocal disease, or close involvement of major vascular structures, which has led international treatment protocols to traditionally favor neoadjuvant cisplatin-based chemotherapy to achieve tumor downstaging and facilitate surgical resection [18,19]. Modern risk assignment increasingly uses unified criteria integrating anatomy (PRETEXT and annotation factors), metastatic status, age, and biomarkers, most prominently within Children’s Hepatic Tumors International Collaboration (CHIC)-derived stratification frameworks which also provide a common language to compare outcomes across collaborative groups [20,21].

AFP remains a central biomarker in HB serving diagnostic, prognostic, and surveillance roles [22,23]. Most tumors exhibit markedly elevated AFP levels, illustrating the fetal origin of the malignant cells. However, recent clinical experience has highlighted important nuances in AFP interpretation. Very low or normal AFP levels at presentation, once considered a uniform marker of aggressive disease, are now recognized as a heterogeneous finding that may reflect biological diversity, diagnostic pitfalls, or alternative tumor entities with overlapping clinical features such as rhabdoid tumors including SMARCB1—altered disease, which have important implications for diagnostic work-up and prognostic counseling. This evolving perspective has reinforced the importance of integrated histopathological and molecular confirmation, particularly in atypical presentations [24].

Risk stratification and treatment planning have increasingly converged toward internationally harmonized systems that combine anatomical staging with clinical and biological variables [19,24,25]. Current protocols incorporate imaging-based liver involvement, vascular extension, metastatic status, patient age, and AFP dynamics to define risk groups and guide therapy intensity [26,27]. While these frameworks have significantly improved outcome comparability and survival rates, they remain largely descriptive and anatomically oriented, offering limited insight into the underlying tumor biology that drives treatment response or resistance.

## 3. Histopathological and Molecular Heterogeneity

HB is an embryonal malignant liver tumor of childhood characterized by a wide range of histologic patterns that recapitulate different stages of hepatic development, ranging from fetal hepatocyte-like differentiation to more primitive embryonal or progenitor-like phenotypes. This intrinsic heterogeneity is a defining feature of HB and has important diagnostic implications, particularly in limited biopsy samples and in post-chemotherapy specimens, where different histological components may coexist in variable proportions. Consequently, careful morphological assessment remains essential, even in the context of increasingly molecularly driven classifications [28,29,30].

### 3.1. Pathological Features of HB and Clinical Correlates

**Figure 1:**
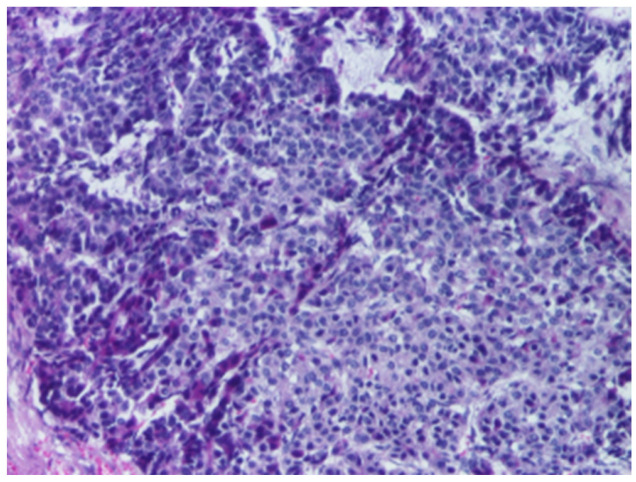
Epithelial hepatoblastoma, hematoxylin-eosin stain (HE) × 200.

**Figure 2:**
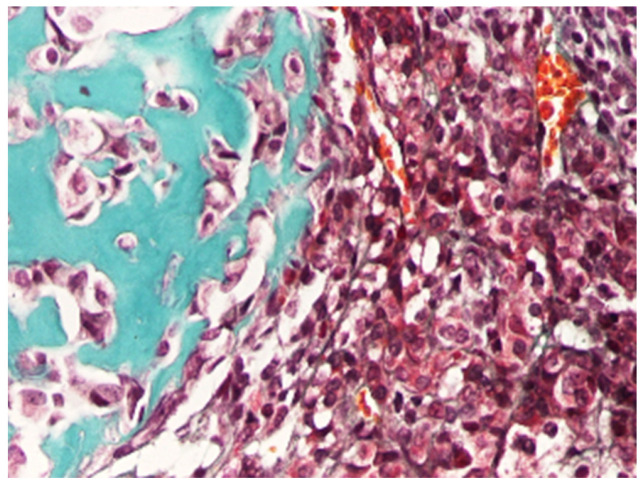
Mixed epithelial–mesenchymal hepatoblastoma, Szekelly stain ×200.

According to the WHO Classification of Paediatric Tumours (5th edition), which builds upon the International Pediatric Liver Tumors Consensus Classification, HB is divided into epithelial (Figure 1) and mixed epithelial–mesenchymal types (Figure 2), with further sub-classification [4,28,30].

**Figure 3:**
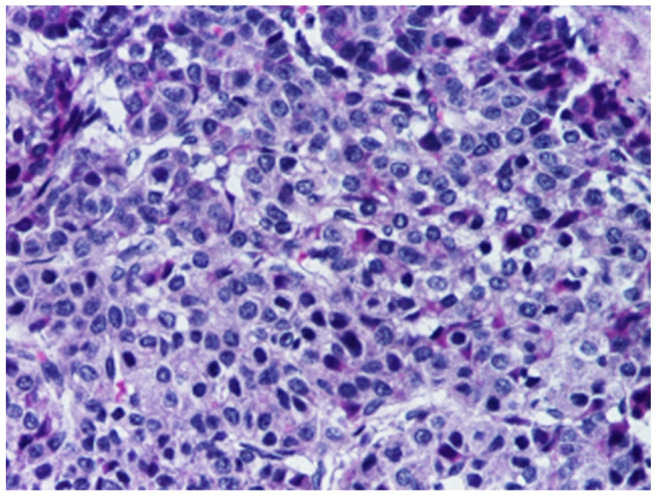
Fetal hepatoblastoma, HE × 200.

**Figure 4:**
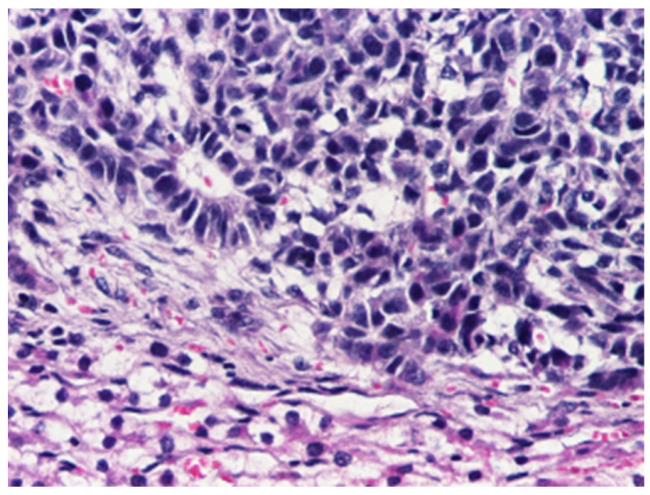
Embryonal hepatoblastoma, HE × 200.

The epithelial category includes several histological patterns, most commonly fetal and embryonal, which frequently coexist within the same tumor [4]. The fetal pattern is composed of relatively uniform cells resembling fetal hepatocytes, arranged in trabecular or sheet-like architectures and typically showing lower mitotic activity (Figure 3), whereas the embryonal pattern consists of smaller, less differentiated cells with a higher nuclear-to-cytoplasmic ratio, increased mitotic activity, and growth in solid, acinar, or rosette-like configurations (Figure 4).

Mixed epithelial–mesenchymal HB is defined by the presence of epithelial components admixed with mesenchymal elements, including fibrous tissue and, in some cases, heterologous differentiation such as osteoid or cartilage. These components are often more readily appreciated in resection specimens than in biopsies, underscoring the importance of adequate sampling and correlation with imaging and clinical findings. Recent pathology reporting recommendations emphasize the need for systematic documentation of the various histological components and their relative proportions, given the recognized intratumoral heterogeneity and its potential clinical relevance (Table 1) [28].

Less frequent variants include the small cell undifferentiated subtype, which is associated with more aggressive behavior and may overlap morphologically with other small round blue cell tumors, as well as macrotrabecular, pleomorphic, and cholangioblastic patterns [4]. The latter shows ductular differentiation and may pose diagnostic challenges, particularly in small biopsies [28].

The differential diagnosis of HB is broad and depends on patient age, clinical context, and morphological features. Pediatric hepatocellular carcinoma represents a key consideration, particularly in older children, while malignant rhabdoid tumor should be excluded in cases with small cell undifferentiated morphology. Other diagnostic considerations include metastatic small round cell tumors and rare primary hepatic mesenchymal neoplasms. Diagnostic challenges are further amplified in post-treatment specimens, where chemotherapy-induced maturation and fibrosis may result in tumor areas that closely resemble non-neoplastic liver parenchyma, increasing the risk of under-recognition of residual disease [30].

Immunohistochemistry is an essential tool in the diagnosis and characterization of HB. Nuclear accumulation of β-catenin is a characteristic finding reflecting activation of the Wnt/β-catenin pathway, one of the central molecular events in HB pathogenesis. Glypican-3 is widely expressed in HB and is particularly useful in highlighting tumor cells and distinguishing them from non-neoplastic liver tissue, particularly in post-therapeutic specimens. AFP expression is variable at the tissue level but remains clinically relevant in correlation with serum levels. Additional markers such as SALL4 support the identification of embryonal components, while cytokeratins (CK7 and CK19) are helpful in demonstrating cholangioblastic differentiation. Evaluation of SMARCB1 (INI1) expression is critical in cases with small cell undifferentiated features to exclude malignant rhabdoid tumor [4,28].

Neoadjuvant chemotherapy induces significant histopathological changes, including necrosis, fibrosis, and varying degrees of tumor maturation, often toward a more fetal-like phenotype with reduced mitotic activity. These therapy-related changes may mask the original tumor architecture and complicate the assessment of viable tumor and resection margins. In this context, correlation with pre-treatment biopsy findings and the use of immunohistochemical markers, particularly glypican-3, are invaluable for accurate interpretation [30].

Pure fetal histology, especially well differentiated, is associated with comparatively favorable prognosis and, in select low-risk cases, may be managed with upfront surgical resection. In contrast, patterns with embryonal or mixed components generally present with more aggressive clinical features and higher proliferative indices. Moreover, certain rare histologic features such as blastemal dominance or small cell morphology have been linked to poorer outcomes, though these associations are currently under investigation [20,24]. Distinct tumor cell populations coexist, including more differentiated fetal-like cells and less differentiated embryonal or stem-like cell clusters characterized by increased proliferative capacity and activation of developmental signaling pathways [8]. These subpopulations may display differential sensitivity to chemotherapy, leading to selective survival of resistant clones under treatment pressure. Over time, clonal selection and expansion of therapy-resistant cell populations may contribute to disease persistence or relapse [29]. This model highlights intratumoral heterogeneity as a key biological driver of variable treatment response and underscores the limitations of uniform therapeutic approaches in pediatric HB.

Modern efforts by the Children’s Hepatic Tumors International Collaboration illustrate ongoing attempts to harmonize histopathological classification with risk stratification frameworks that incorporate age, AFP levels, metastasis status, and PRETEXT staging [4,29]. Importantly, most HBs are composed of mixed histologic elements, rather than pure subtypes, indicating intratumoral diversity at the cellular level. This mosaic pattern suggests that distinct cellular populations within the same tumor may differ in differentiation status and in their contributions to chemotherapeutic sensitivity or resistance [2,30].

### 3.2. Molecular Landscape and Biological Diversity

Despite a relatively low mutational burden compared with adult hepatic tumors, HB consistently shows recurrent alterations involving developmental regulatory pathways (Table 2) [2,30].

The Wnt/β-catenin pathway is the dominant molecular driver, with activating mutations in catenin beta 1 (CTNNB1) observed in most cases and serving as a hallmark of oncogenic signaling in this disease [2,31,32].

Beyond Wnt signaling, recent transcriptomic and spatial analyses have delineated distinct molecular signatures corresponding to fetal-like and embryonal-like tumor cell populations, reflecting underlying developmental hierarchies retained within tumors. These studies reveal a conserved transcriptomic signature among fetal tumor cells across patients, whereas embryonal-enriched populations show greater inter-patient heterogeneity, underscoring the biological diversity of tumor cell states [31,33].

Additional signaling networks implicated in HB include the Hippo–YAP axis, which modulates cellular proliferation and survival, as well as metabolic regulators and pathways involved in stemness and differentiation. Epigenetic mechanisms, including DNA methylation alterations and chromatin accessibility changes, further contribute to tumor diversity and may underlie differential gene expression programs observed across subgroups [2].

Notch signaling has emerged as an important regulator of cell fate control in liver development and is increasingly implicated in HB pathogenesis. Aberrant activation of the Notch pathway contributes to the maintenance of progenitor-like tumor cell populations, promotes cellular plasticity, and may interact with other developmental pathways such as Wnt/β-catenin and Hippo–YAP. These interactions support tumor growth, intratumoral heterogeneity, and resistance to therapy. Even if not yet routinely integrated into clinical risk stratification, Notch signaling represents a potential therapeutic target and a biologically relevant component of HB progression [34].

Although these pathways converge on growth and survival, they influence hepatoblastoma biology through partially distinct axes. Aberrant Wnt/β-catenin signaling is closely linked to maintaining progenitor-like transcriptional programs and altered differentiation paths [33,35]. Hippo pathway inactivation with YAP/TAZ activation promotes regenerative-like proliferative states and supports expansion of less differentiated compartments and microenvironmental crosstalk [36,37]. The PI3K/AKT/mTOR axis acts as a key metabolic and survival integrator and may modulate therapy response by enhancing stress adaptation and growth under fluctuating nutrient/oxygen conditions. The relative dominance of these programs can vary across tumors and even across subclones, supporting a pathway-based explanation for pathological diversity and variable chemosensitivity [38,39,40].

Emerging single-cell and spatial profiling techniques have begun to elucidate the complex interplay between tumor cell states and the microenvironment, revealing heterogeneous expression of transcription factors, metabolic genes, and lineage markers that are not evident in analyses of mixed cell populations. These insights emphasize the existence of molecularly defined cell populations within hepatoblastoma that may mediate distinct clinical behaviors and treatment responses [31,33].

### 3.3. Clinical Implications of Heterogeneity

Recent single-cell and clonal analyses support that HB is not a uniform entity, but rather a mixture of tumor cell states that recapitulate developmental trajectories. Distinct populations resembling fetal-like differentiated hepatocytic programs coexist with embryonal/stem-like progenitor-like states, often accompanied by variable stromal, endothelial, and immune microenvironmental components [25,41]. Clinically, this heterogeneity is relevant because tumors with embryonal/progenitor-like features tend to grow faster, are less differentiated, and respond less well to therapy, while tumors with fetal-like features are more differentiated and may respond better to chemotherapy [33]. For that reason, baseline sampling and post-therapy specimens may capture different dominant cellular states, which complicates histology–risk correlations, response assessment, and the identification of actionable targets [42].

Accordingly, morphological and molecular heterogeneity is of direct clinical relevance. Fetal-differentiated tumors with concordant molecular profiles may respond better to chemotherapy and carry a more favorable prognosis, while embryonal-like or stem-enriched signatures are commonly associated with aggressive disease and a higher likelihood of relapse as stated before [20] (Table 3).

Importantly, traditional risk stratification systems based on imaging and clinical parameters do not fully capture this biological diversity and thus may overlook key determinants of therapeutic response and long-term outcome [29,43].

Integrating histopathological classification with high-resolution molecular profiling holds promise for more precise risk stratification and may inform personalized treatment approaches. For example, identification of dominant embryonal transcriptional programs or stemness-associated molecular signatures could guide the use of targeted agents or novel therapeutic modalities in high-risk subgroups, whereas tumors with well-differentiated features might be considered for therapy de-escalation trials [33,44].

Conventional risk stratification relies primarily on anatomical extent (PRETEXT staging), serum AFP levels, metastatic status, and surgical resectability [20]. While effective in improving overall survival, this approach does not fully capture the biological heterogeneity underlying variable treatment response and relapse risk. The proposed model integrates histopathological features with key molecular determinants, including activation of developmental signaling pathways, stemness-associated programs, and proliferative signatures [1,2]. Incorporating molecular information into risk assessment may enable refined therapeutic stratification, guide surgical planning, identify candidates for treatment intensification or de-escalation, and support the rational introduction of targeted and innovative therapies in pediatric HB [2,9,45].

Together, these findings highlight that HB is not a uniform disease but a constellation of biologically diverse subtypes. Embracing this complexity through integrated histological and molecular paradigms is essential for advancing precision oncology in pediatric liver cancer.

## 4. Developmental Molecular Mechanisms

Pediatric HB arises from disrupted programs of embryonic liver development, reflecting the persistence and dysregulation of signaling pathways that normally orchestrate hepatic organogenesis and growth control. At the heart of this process are a set of interlinked molecular circuits, most prominently Wnt/β-catenin, Hippo–YAP/TAZ, insulin-like growth factor (IGF), and PI3K/AKT/mTOR pathways which together drive malignant transformation, sustain proliferative signaling, and shape tumor cell phenotypes. Understanding these developmental mechanisms provides crucial insights into the biology of HB and lays the foundation for targeted interventions [2,46].

**Figure 5:**
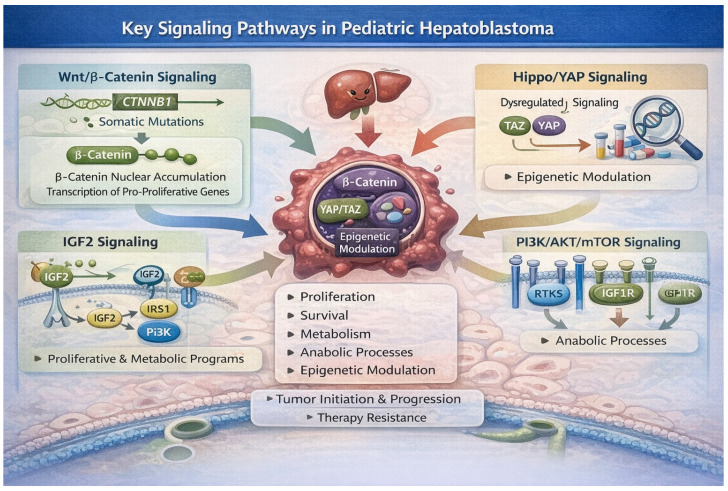
Developmental molecular integrated circuit underlying pediatric hepatoblastoma. This schematic illustrates key signaling pathways that govern liver development and are co-opted in hepatoblastoma pathogenesis.

Canonical Wnt/β-catenin activation through somatic mutations in CTNNB1 leads to nuclear β-catenin accumulation and transcriptional activation of pro-proliferative genes. Dysregulated Hippo signaling permits YAP/TAZ nuclear activity, cooperating with β-catenin to enhance growth and survival [47]. IGF2-mediated signaling supports proliferative and metabolic programs, while PI3K/AKT/mTOR integrates growth factor cues and sustains anabolic processes. Epigenetic modulators further shape transcriptional landscapes, reinforcing cell identity and phenotypic heterogeneity [48]. The interconnected nature of these pathways drives tumor initiation, progression, and therapy resistance in pediatric HB (Figure 5).

### 4.1. Wnt/β-Catenin Signaling: Core Oncogenic Driver

Activation of the canonical Wnt/β-catenin pathway represents the most frequent and defining molecular alteration in HB. The pathway is triggered when Wnt ligands bind Frizzled receptors and LRP5/6 co-receptors, leading to stabilization and nuclear translocation of β-catenin, which in turn complexes with TCF/LEF transcription factors to promote expression of growth and survival genes [19,48]. Somatic mutations in CTNNB1, particularly within exon 3, impair the proteasomal degradation of β-catenin, resulting in constitutive pathway activation in most tumors. Nuclear β-catenin accumulation is correlated with embryonal histology and aggressive clinical behavior, underscoring its central role in HB initiation and maintenance [49].

Although Wnt/β-catenin activation is necessary, it is not sufficient to drive malignant transformation alone in experimental systems, indicating that cooperation with additional pathways is required to fully recapitulate the HB phenotype [40].

### 4.2. Hippo–YAP/TAZ Pathway: Growth Control and Tumor Progression

The Hippo signaling cascade functions as a key regulator of organ size, balancing proliferation and apoptosis during liver development and regeneration. Under active Hippo signaling, kinase cascades restrain the activity of transcriptional co-activators YAP and TAZ. When Hippo signaling is suppressed or impaired, unphosphorylated YAP/TAZ translocate to the nucleus and drive expression of genes promoting cell proliferation and survival. Experimental evidence supports a model in which concurrent activation of YAP/TAZ with β-catenin accelerates HB formation and enhances tumor growth, pointing to a mechanistic synergy between these pathways in pediatric liver oncogenesis [40,48].

Single-cell and spatial analyses further indicate that subsets of HB cells exhibit high levels of YAP/TAZ concomitant with stem-like transcriptional programs, suggesting that dysregulated Hippo signaling may contribute to intratumoral heterogeneity and therapy resistance [40,48].

### 4.3. IGF Signaling and Metabolic Regulation

Insulin-like growth factor signaling bridges developmental growth control and oncogenic proliferation. Overexpression of IGF2, often driven by imprinting defects or upstream pathway crosstalk, sustains proliferative and anti-apoptotic signals within tumor cells [38]. Recent single-cell analyses have highlighted IGF2-driven clusters of malignant hepatoblast-like cells that display enhanced proliferative capacity and metabolic reprogramming, implicating IGF2 not only as a growth factor but also as a determinant of cellular identity and aggressiveness within the tumor microenvironment [45].

### 4.4. PI3K/AKT/mTOR Axis and Integrative Growth Signaling

The PI3K/AKT/mTOR pathway is a pivotal nexus integrating growth factor signals with cellular metabolism. Aberrant activation of this cascade has been documented in HB and is functionally linked with both Wnt/β-catenin and YAP signaling [48]. Preclinical models driven by co-activation of β-catenin and YAP demonstrate upregulation of mTOR activity, and pharmacologic inhibition of mTOR reduces tumor burden and alters differentiation states toward a less aggressive phenotype. These findings suggest that mTOR functions downstream of developmental signals to sustain proliferative and survival programs [50].

### 4.5. Epigenetic and Transcriptional Modulation

Beyond canonical signaling pathways, HB exhibits complex layers of epigenetic regulation that modulate gene expression and cellular differentiation [1,2]. Global patterns of DNA hypomethylation, focal hypermethylation of tumor suppressor loci, and alterations in chromatin accessibility contribute to transcriptional programs favoring proliferation and stem-like states. Integration of epigenomic and transcriptomic profiles has identified molecular subgroups with distinct differentiation states and prognostic implications, indicating that epigenetic landscapes are integral to the developmental arrest characteristic of HB [2,31].

Together, these pathways operate not as isolated modules but as an interconnected network that recapitulates, and in some instances assumes, normal hepatic developmental programs. Dysregulation of these networks underlies the proliferative advantage, cellular plasticity, and adaptive responses observed in HB [8,9]. While CTNNB1 mutations represent key initiating events in HB, accumulating evidence suggests that tumor behavior and treatment response are further influenced by coordinated interactions with Hippo–YAP signaling, IGF and mTOR axis activity, and epigenetic regulatory programs [6,32]. Targeting these integrated developmental circuits represents a promising strategy to overcome resistance to conventional therapy and to tailor treatment to specific biological vulnerabilities.

## 5. Tumor Stemness and Microenvironment

Beyond genetic and signaling alterations, HB is increasingly recognized as a tumor sustained by developmental immaturity, cellular plasticity, and a permissive microenvironment. Accumulating evidence indicates that stem-like tumor cell populations and a uniquely configured immune and vascular niche play central roles in tumor maintenance, therapeutic resistance, and disease recurrence [46,47]. These features further reinforce the concept of HB as a developmentally driven malignancy rather than a conventional solid tumor.

### 5.1. Hepatic Progenitor Cells and the Cancer Stem Cell Phenotype

HB recapitulates phenotypic features of fetal hepatic progenitor cells, reflecting an arrest in normal hepatocytic differentiation. At the cellular level, subsets of tumor cells exhibit expression of progenitor and stemness-associated markers, most notably epithelial cell adhesion molecule (EpCAM) and CD133 (PROM1). These markers are characteristic of immature hepatoblasts during liver development and are retained in distinct tumor cell populations within HB [51].

EpCAM-positive and CD133-positive cells have been shown to possess enhanced clonogenic capacity, increased proliferative potential, and resistance to cytotoxic stress, consistent with a cancer stem cell–like phenotype [52]. Recent transcriptomic and single-cell profiling studies have demonstrated that these populations are enriched for developmental signaling pathways, including Wnt/β-catenin and YAP-associated transcriptional programs, positioning stem-like cells at the intersection of developmental signaling and oncogenic persistence. Importantly, these cells often coexist with more differentiated tumor populations, contributing to intratumoral heterogeneity and functional diversity [46,47].

The presence of stem-like tumor cell compartments has significant therapeutic implications. Experimental models suggest that EpCAM- and CD133-expressing cells are less sensitive to conventional chemotherapy, surviving treatment and subsequently repopulating the tumor [2,53,54]. This selective pressure may explain why radiographic and biochemical responses do not always translate into durable remission, particularly in high-risk or relapsed HB [55]. Thus, tumor stemness emerges as a key biological driver of chemoresistance and disease recurrence.

### 5.2. Immune and Vascular Microenvironment

In parallel with intrinsic tumor cell programs, the HB microenvironment plays an essential role in shaping tumor behavior. Angiogenesis is a prominent feature of HB, driven in part by hypoxia-induced signaling and vascular endothelial growth factor (VEGF) expression [56]. Hypoxic regions within large or poorly perfused tumors activate hypoxia-inducible factors, which in turn promote VEGF-mediated neovascularization. While this vascular network supports tumor growth and survival, it is often structurally and functionally abnormal, contributing to heterogeneous drug delivery and variable therapeutic efficacy [46].

The immune microenvironment of HB is increasingly characterized as immunologically quiescent or “immune-cold.” Compared with adult liver cancers, HB generally exhibits low tumor mutational burden and limited neoantigen expression, which reduces spontaneous immune recognition [42,57]. In addition, recent immunoprofiling studies have demonstrated sparse infiltration of cytotoxic T lymphocytes and a predominance of immunosuppressive myeloid populations, including tumor-associated macrophages with protumoral phenotypes [58]. These features collectively contribute to immune evasion and may underlie the limited efficacy observed with immune checkpoint inhibitors in early clinical experiences [59].

Importantly, the immune and vascular components of the tumor microenvironment are not passive spectators but actively interact with stem-like tumor cells [57]. Hypoxia has been shown to reinforce stemness-associated transcriptional programs, while paracrine signaling within the microenvironment may protect progenitor-like cells from immune-mediated clearance. This bidirectional crosstalk further stabilizes aggressive tumor phenotypes and limits the effectiveness of uniform treatment approaches [57,58].

### 5.3. Biological and Therapeutic Implications

The convergence of tumor stemness and a permissive microenvironment provides a unifying framework for understanding heterogeneity, treatment resistance, and relapse in pediatric HB. Stem-like tumor cell populations, supported by hypoxia, angiogenesis, and immune evasion, represent a biologically resilient compartment that is insufficiently addressed by conventional chemotherapy alone. These insights suggest that effective therapeutic strategies may require combined targeting of intrinsic stemness programs and extrinsic microenvironmental factors [42,45].

By integrating tumor cell plasticity with immune and vascular context, this perspective underscores the need for biologically informed therapeutic innovation, setting the stage for the exploration of targeted, anti-angiogenic, and immune-modulatory approaches discussed in subsequent sections.

## 6. Current Therapeutic Strategies and Limitations

The current therapeutic approach to pediatric HB has achieved remarkable success, with overall survival exceeding 80% in standard-risk patients. These outcomes are largely attributable to cisplatin-based chemotherapy, improved surgical techniques, and the expanded use of liver transplantation for unresectable tumors [18]. Nevertheless, current treatment strategies remain predominantly guided by anatomical and clinical parameters, with limited incorporation of tumor biology into therapeutic decision-making, a limitation that becomes increasingly evident in high-risk and relapsed disease [5].

### 6.1. Risk Stratification: Strengths and Structural Limitations

Current risk stratification in pediatric HB remains primarily clinico-radiological, with anatomy-based imaging serving as the central determinant of therapeutic allocation. The PRETEXT system, together with its standardized annotation factors such as capturing vascular involvement, extrahepatic spread, multifocality, tumor rupture, nodal involvement, and distant metastases, provides a robust and reproducible framework for baseline evaluation of tumor burden and for guiding resectability planning across institutions [60]. While collaborative groups have historically applied different risk schemas, reflecting variations in upfront resectability assessment and treatment intensity, efforts toward harmonization have progressed substantially. CHIC established an evidence-based, globally unified approach to risk-stratified staging (CHIC-HS), integrating PRETEXT extent with key clinical variables to enable more consistent international risk grouping and cross-trial comparability [19,61]. Current protocols increasingly rely on such harmonized stratification models as the structural basis for treatment adaptation, although routine incorporation of molecular features into formal risk classification remains an evolving, non-uniform practice [19].

These systems have been instrumental in guiding neoadjuvant chemotherapy, surgical planning, and transplant referral. Nevertheless, they are inherently static and anatomy-focused, describing tumor distribution rather than biological aggressiveness [62].

Importantly, tumors with similar PRETEXT stages may exhibit markedly different responses to identical treatment regimens, reflecting underlying molecular and cellular diversity. Current stratification does not formally account for histological differentiation gradients, stem-like tumor cell populations, or activation of specific oncogenic pathways [19,63]. As a result, biologically aggressive tumors may receive suboptimal therapy, whereas indolent lesions may be subjected to unnecessary toxicity.

### 6.2. Chemotherapy: Efficacy at the Cost of Selective Pressure

Cisplatin remains the most active cytotoxic agent in HB and is the backbone of both standard- and high-risk treatment regimens. In standard-risk HB, cisplatin-based therapy, including cisplatin monotherapy evaluated in selected protocols, has been associated with high survival rates while aiming to reduce treatment-related toxicity [64]; however, outcomes for high-risk patients remain suboptimal despite treatment intensification, with relapse and chemoresistance continuing to represent major clinical challenges. Therefore, its success must be viewed alongside its biological limitations. Cytotoxic chemotherapy primarily targets rapidly proliferating cells, which may preferentially eliminate differentiated tumor populations while sparing slower-cycling, stem-like cells [65].

Such selective pressure can reshape tumor composition, enriching for chemotherapy-resistant cell subsets that drive persistence or relapse. In clinical practice, this is reflected by scenarios in which early radiologic regression and declining AFP levels are not accompanied by sustained disease control [8]. Furthermore, cumulative toxicity, most notably ototoxicity and nephrotoxicity, remains a significant concern, particularly given the young age of affected patients and the importance of long-term quality of life [65]. Therefore, Cisplatin-based regimens remain foundational in HB treatment, achieving excellent outcomes in standard-risk patients; however, high-risk disease continues to register comparatively poor event-free and overall survival despite intensive chemotherapy strategies, illustrating the limitations of current systemic therapy approaches [66].

### 6.3. Surgery and Liver Transplantation: Curative Strategies with Limited Biological Integration

This section summarizes key surgical principles and decision-making in hepatoblastoma management such as resectability assessment, timing relative to chemotherapy, and indications for liver transplantation. Given the technical complexity and variability of pediatric liver surgery and transplantation, a comprehensive description of operative maneuvers, intraoperative troubleshooting, and technique-specific nuances would require dedicated surgical and visual resources and is therefore beyond the scope of the present narrative review.

Surgical resection remains the only definitive curative modality for HB. Continued advances in operative strategy, perioperative management, interventional radiology support, and pediatric liver transplantation have substantially increased the proportion of patients who can achieve complete tumor clearance, including those with initially extensive disease [67]. In routine practice, however, surgical decision-making is still driven predominantly by clinic-radiological criteria, such as tumor topography, vascular proximity or involvement, multifocality, and estimates of future liver remnant rather than by tumor-intrinsic biology [68]. This anatomy-centered paradigm is essential for operative safety, yet it provides limited information on the likelihood of occult dissemination, microscopic residual disease, or early relapse.

Recent advances have significantly refined the surgical management of hepatoblastoma (HB), with curative outcomes remaining critically dependent on the achievement of complete (R0) resection. In patients with chemotherapy-resistant or metastatic disease, aggressive surgical strategies, including extended hepatectomy, liver transplantation (LT), and resection of residual metastatic lesions, are essential and may confer favorable long-term survival, even in the presence of persistent pulmonary metastases [69].

Hepatic resections are performed according to established anatomical principles (Brisbane 2000 Terminology) and may include anatomical or non-anatomical approaches, via open or minimally invasive techniques. Intraoperative vascular control is a key component and is typically achieved using the Pringle maneuver or, in complex resections, total vascular exclusion with suprahepatic and infrahepatic inferior vena cava control [70].

Preoperative planning relies increasingly on three-dimensional (3D) reconstruction and virtual hepatectomy, enabling precise assessment of tumor extent, vascular relationships, and future liver remnant (FLR). Diffusion-weighted MRI may improve detection of satellite lesions. Intraoperatively, indocyanine green fluorescence imaging and augmented reality navigation facilitate tumor localization and margin assessment, particularly in minimally invasive approaches [69].

Minimally invasive liver resection is reserved for selected patients, typically with tumors located in anterolateral segments (S2–S6), and includes wedge resections, segmentectomies, and left lateral sectionectomy. Robotic-assisted hepatectomy remains in early approval but offers potential advantages in terms of dexterity, precision, and reduced intraoperative blood loss [71].

In patients with insufficient FLR, strategies to induce hypertrophy, such as associating liver partition and portal vein ligation for staged hepatectomy, may allow safe completion of extended resections. For centrally located or unresectable tumors (POST-TEXT III–IV), primary LT remains the standard curative option, even in cases with apparent radiological response but potential residual microscopic disease [69].

Advanced techniques derived from transplant surgery, including vascular resection and reconstruction, ante-situm and ex vivo liver resection with autotransplantation, have been successfully applied in highly selected cases, extending resectability beyond conventional limits [72,73].

Pulmonary metastases are present in approximately 20% of patients at diagnosis. In cases of incomplete response to chemotherapy, surgical metastasectomy, including repeated thoracotomies, remains a key component of curative-intent treatment [69].

From a biological standpoint, surgery does not differentiate between tumors with favorable versus high-risk molecular behavior. Two lesions categorized as technically resectable may nonetheless exhibit markedly different propensities for recurrence, reflecting differences in differentiation status, stemness-associated programs, pathway activation, and microenvironmental adaptation [69]. Consequently, a complete (R0) resection may be curative for one patient but insufficient for another whose tumor biology favors early regrowth or metastatic outgrowth, even in the absence of overt residual disease. This mismatch may contribute to the persistent subset of patients who relapse despite apparently optimal local control [67].

Similarly, LT provides a life-saving option for unresectable HB and has transformed outcomes in selected patients, yet it does not take tumor biology into account [74]. Current transplant candidacy is primarily determined by anatomical unresectability and response to neoadjuvant therapy rather than by validated biomarkers predicting post-transplant recurrence. Moreover, the need for long-term immunosuppression introduces a unique biological context in which minimal residual disease could potentially expand, underscoring the importance of relapse-risk estimation beyond imaging findings alone [75,76]. In this setting, the absence of biology-informed criteria also limits rational tailoring of peri-transplant systemic therapy, including escalation for tumors with aggressive molecular signatures versus de-escalation for biologically indolent disease.

Looking forward, integrating molecular and immunophenotypic data into surgical and transplant algorithms represents a critical step toward truly personalized local therapy. The field is increasingly moving toward risk-adapted strategies that do not treat resectability as a purely technical endpoint, but rather as one component of a broader framework incorporating relapse biology, treatment sensitivity, and potential for therapy-related toxicity. Such integration may ultimately refine timing of surgery, optimize selection for upfront transplantation, and support individualized perioperative systemic approaches aimed at minimizing recurrence while avoiding overtreatment [77].

From a surgical perspective, data standardization plays a critical role in ensuring consistent and safe decision-making, yet remains insufficiently integrated with emerging biological insights. Standardized imaging acquisition and reporting, particularly through consistent application of PRETEXT/POSTTEXT staging and annotation factors, are essential for reproducible assessment of resectability and surgical planning [60]. In addition, volumetric analyses, including estimation of the FLR, require harmonized methodologies to guide the extent of resection and reduce the risk of postoperative liver failure. Standardized pathological evaluation following chemotherapy, including tumor necrosis/regression, vascular invasion, and margin status (R0/R1/R2), is equally important for postoperative risk stratification and outcome comparison across centers [2]. Despite these advances in clinical standardization, integration of molecular risk profiles into surgical decision-making remains limited, highlighting an important gap between biological understanding and operative strategy [78]. Future efforts should aim to bridge this gap, enabling more personalized approaches, including optimized selection between extended resection and LT.

### 6.4. From Limited Molecular Integration to a Biology-Informed Therapeutic Paradigm

Despite growing insight into the molecular foundations of HB, routine clinical management remains disconnected from molecular profiling. Outside of rare diagnostic scenarios, molecular data are seldom used to guide treatment intensity, select therapeutic agents, or inform surgical timing. This disconnect reflects both the historical success of clinically driven protocols and the lack of validated biomarkers with immediate therapeutic implications [2].

However, as survival continues to improve, the limitations of this approach are becoming increasingly evident. Relapsed and refractory HB still carries a poor prognosis, underscoring the need for therapeutic strategies that extend beyond further escalation of cytotoxic chemotherapy [79,80,81]. Without integration of biological vulnerability and resistance mechanisms, further gains in outcome are likely to stagnate.

The current therapeutic framework for HB excels at achieving macroscopic tumor control but is less effective at addressing microscopic biological resilience. The absence of molecular stratification perpetuates a “one-size-fits-most” approach that inadequately reflects tumor diversity [82]. Recognizing these limitations is a critical step toward redefining treatment paradigms.

Future strategies must reconcile anatomical feasibility with biological behavior, integrating molecular and cellular insights into decisions regarding chemotherapy intensity, surgical timing, and transplant candidacy. Such a shift would enable rational de-escalation in biologically favorable tumors while prioritizing innovative or intensified approaches for high-risk disease.

## 7. Innovative and Emerging Therapeutic Approaches

The recognition that conventional, anatomy-centered decision-making treatment strategies inadequately address the biological complexity of HB has driven the exploration of innovative therapeutic approaches [83,84]. These emerging strategies aim to exploit specific molecular vulnerabilities, disrupt tumor–microenvironment interactions, and overcome resistance mechanisms rooted in developmental signaling and cellular plasticity. Although most novel therapies remain in early clinical or preclinical stages, they represent a critical step toward biologically informed treatment paradigms [2,33,42].

### 7.1. Targeted Therapies

Table 4 provides a proposed biomarker-informed combination strategy in HB, clarifying how these strategies may interface with current standard-of-care pathways.

#### 7.1.1. mTOR Inhibitors

The PI3K/AKT/mTOR axis occupies a central position at the intersection of growth factor signaling, metabolism, and cell survival in HB [9]. Preclinical studies have demonstrated that mTOR activation is frequently downstream of aberrant Wnt/β-catenin and YAP signaling, positioning mTOR as a potential integrative therapeutic target [50]. Pharmacologic inhibition of mTOR has been shown to reduce tumor growth, impair anabolic metabolism, and modulate differentiation states in experimental HB models [6,85].

Clinically, mTOR inhibitors such as everolimus have been explored primarily in relapsed or refractory settings, often within compassionate-use frameworks. While objective responses have been limited, disease stabilization in selected cases suggests that mTOR inhibition may exert cytostatic rather than cytotoxic effects. These observations imply that mTOR inhibitors may be more effective as part of combination strategies, particularly when used to suppress stem-like tumor cell populations that survive conventional chemotherapy [6,7].

#### 7.1.2. Targeting Wnt/β-Catenin and Hippo–YAP Signaling: Biological and Practical Challenges

Given the near-universal involvement of Wnt/β-catenin signaling in HB, direct pathway inhibition represents an attractive therapeutic concept. However, clinical translation has been hindered by substantial challenges [2]. Wnt signaling is essential for normal tissue homeostasis, particularly in pediatric patients, raising concerns regarding systemic toxicity and developmental side effects. Moreover, β-catenin functions both as a transcriptional regulator and as a structural component of adherens junctions, complicating selective pharmacologic targeting [6].

Similarly, the Hippo–YAP pathway has emerged as a critical driver of tumor growth and stemness, especially in cooperation with β-catenin. While YAP inhibition has shown promise in preclinical systems, the lack of highly specific, clinically validated YAP inhibitors remains a major obstacle [33]. These challenges underscore the need for indirect targeting strategies, such as disrupting pathway crosstalk or downstream effectors, rather than attempting to block master regulators outright [15].

### 7.2. Anti-Angiogenic Strategies

Angiogenesis represents a hallmark of HB, driven by hypoxia and sustained VEGF signaling. This dependency has prompted interest in anti-angiogenic therapies as adjuncts to conventional treatment. Agents such as bevacizumab and multikinase inhibitors like sorafenib have demonstrated anti-tumor activity in preclinical models and isolated clinical reports [46,47].

Anti-angiogenic therapy may exert multifaceted effects, including normalization of abnormal tumor vasculature, reduction in hypoxia, and improved delivery of cytotoxic agents. However, clinical experience to date suggests that anti-angiogenic agents alone are insufficient to achieve durable tumor control. Moreover, adaptive resistance mechanisms, including alternative pro-angiogenic signaling and increased tumor invasiveness, may limit long-term efficacy. These observations support the rationale for integrating anti-angiogenic strategies into combination regimens rather than deploying them as monotherapies [2,81].

### 7.3. Immunotherapy and Epigenetic Modulation

Despite transformative success in other pediatric and adult malignancies, immune checkpoint inhibition has shown limited efficacy in HB. This resistance reflects several intrinsic biological features of the disease [1,57]. HB typically exhibits a low tumor mutational burden, resulting in limited neoantigen generation and reduced immunogenicity. In addition, the tumor microenvironment is characterized by sparse infiltration of cytotoxic T lymphocytes and enrichment of immunosuppressive myeloid populations, reinforcing an immune-cold phenotype [1,6].

Furthermore, developmental signaling pathways that sustain tumor stemness may actively suppress immune recognition, creating an environment in which immune checkpoint blockade alone is insufficient to elicit robust anti-tumor responses [57]. These factors collectively explain why immunotherapy has not yet achieved meaningful clinical impact in HB.

Epigenetic dysregulation plays a pivotal role in maintaining the undifferentiated, stem-like state of HB cells and in shaping their interaction with the immune system [1,9,45]. DNA methylation and histone modification patterns can silence antigen presentation machinery and immune-stimulatory pathways, further contributing to immune evasion [86].

Epigenetic therapies, including histone deacetylase inhibitors and DNA methylation modulators, have emerged as potential tools to reprogram tumor cells toward a more differentiated and immunogenic phenotype. By altering chromatin accessibility and gene expression, these agents may enhance tumor antigen presentation, sensitize tumors to immune-mediated killing, and synergize with immunotherapeutic approaches. Although clinical data in HB remain limited, this strategy offers a biologically rational avenue to convert immune-cold tumors into immune-responsive targets [6].

Collectively, innovative therapeutic approaches in HB highlight both promise and complexity. Targeted agents, anti-angiogenic therapies, immunotherapy, and epigenetic modulation each address specific aspects of tumor biology but are unlikely to succeed as standalone interventions. Instead, their greatest potential lies in rational combinations tailored to molecular and microenvironmental contexts [2]. These strategies represent a shift from empiric treatment escalation toward precision-guided intervention, setting the stage for future clinical trials designed around biological vulnerability rather than anatomical extent alone [6].

Given the rarity of hepatoblastoma, much of the evidence for targeted and immune strategies remains early-phase or hypothesis-generating; therefore, we explicitly distinguish evidence-supported standards of care (cooperative-group trials) from investigational approaches (preclinical rationale, small cohorts, or ongoing trials) when proposing combination strategies. Key trials and high-value evidence sources informing emerging or biomarker-enabled strategies are summarized in Table 5, with clear separation of evidence-supported backbones from hypothesis-generating therapeutic proposals.

## 8. Biomarkers and Liquid Biopsy

The increasing recognition of biological heterogeneity in HB has exposed the limitations of conventional biomarkers, particularly when used in isolation. While AFP remains indispensable for diagnosis and treatment monitoring, it provides limited insight into tumor biology, clonal dynamics, or emerging resistance [94,95]. In this context, liquid biopsy approaches have gained attention as minimally invasive tools capable of capturing real-time molecular information and complementing traditional clinical assessment [96,97].

### 8.1. Circulating Tumor DNA (ctDNA)

Circulating tumor DNA represents fragmented tumor-derived genetic material released into the bloodstream through apoptosis, necrosis, or active secretion. In HB, ctDNA analysis offers the potential to detect tumor-specific genetic alterations, including pathway-defining mutations, without the need for repeated tissue biopsies. This is particularly relevant in pediatric patients, where invasive sampling is often limited [98].

Beyond diagnostic applications, ctDNA may enable dynamic monitoring of treatment response and early detection of minimal residual disease. Changes in ctDNA burden during therapy could reflect biological response more sensitively than imaging or AFP kinetics alone. Importantly, ctDNA profiling may also reveal clonal evolution under therapeutic pressure, providing insights into mechanisms of resistance and relapse that are otherwise inaccessible through conventional monitoring [32,99].

### 8.2. MicroRNAs as Functional Biomarkers

MicroRNAs (miRNAs) have emerged as key post-transcriptional regulators of gene expression and play significant roles in liver development, differentiation, and oncogenesis [100]. In HB, dysregulated miRNA networks reflect the persistence of fetal transcriptional programs and the suppression of differentiation pathways. Circulating miRNAs are particularly attractive biomarkers due to their stability in blood and their capacity to mirror tumor biology [101].

Distinct miRNA expression patterns have been associated with aggressive disease features, stem-like phenotypes, and therapeutic resistance. As such, miRNAs may serve not only as diagnostic or prognostic markers but also as functional indicators of pathway activation. Integrating miRNA profiles with other molecular data could enhance risk stratification and identify patients who may benefit from targeted or combination therapies [102].

### 8.3. DNA Methylation Profiling

Epigenetic profiling has added a further dimension to biomarker discovery in HB. DNA methylation patterns provide stable, cell-of-origin–related signatures that capture developmental arrest and lineage characteristics. Recent studies suggest that methylation-based classification can distinguish biologically distinct tumor subsets and may outperform single-gene or mutation-based approaches in predicting clinical behavior [103].

Methylation profiling also offers potential diagnostic utility in cases with atypical histology or low AFP levels, where conventional classification is challenging. Moreover, because epigenetic states are reversible, methylation signatures may help identify tumors responsive to epigenetic therapies, linking biomarker discovery directly to therapeutic innovation [8].

Liquid biopsy approaches represent a paradigm shift from static, tissue-based assessment toward dynamic, biology-driven monitoring. While their clinical implementation in HB remains in early stages, ctDNA, miRNA, and methylation profiling collectively hold promise for refining risk stratification, guiding treatment adaptation, and enabling earlier intervention in relapsed disease. Their greatest value is likely to emerge when integrated with clinical parameters and functional models rather than used as standalone tools [100,104].

## 9. Translational Models and Precision Medicine

Bridging molecular discovery and clinical application in HB requires robust experimental systems that faithfully recapitulate tumor biology. Traditional cell lines, while valuable, fail to capture the developmental context, cellular heterogeneity, and microenvironmental interactions characteristic of pediatric liver tumors [105]. In response, a new generation of translational models has emerged, supporting the transition toward precision medicine [106].

### 9.1. Patient-Derived Organoids

Organoids generated from HB tissue preserve key architectural, molecular, and functional features of the original tumor, including differentiation states and pathway activation patterns. These three-dimensional cultures provide a powerful platform for studying tumor biology in a patient-specific context and for testing therapeutic vulnerabilities in vitro [105].

Importantly, organoids retain intratumoral heterogeneity, enabling exploration of differential drug sensitivity among distinct cellular populations. This feature makes them particularly suited for investigating stem-like compartments and treatment resistance mechanisms [107]. In the future, organoid-based drug screening could inform personalized therapeutic strategies, especially in relapsed or refractory disease.

### 9.2. Patient-Derived Xenografts (PDX)

Patient-derived xenograft models offer complementary advantages by maintaining tumor architecture and biological behavior within an in vivo environment. PDX models have been instrumental in validating oncogenic drivers, assessing drug efficacy, and studying tumor progression under therapeutic pressure [108]. In HB, these models have demonstrated that pathway-targeted therapies may produce heterogeneous responses depending on the molecular context of the tumor [109]. However, PDX models are resource-intensive and limited by the absence of a fully functional immune system in host animals, constraining their utility for immunotherapy studies. Despite these limitations, they remain a critical tool for preclinical validation of novel therapeutic approaches [110,111].

### 9.3. CRISPR-Based Functional Genomics

CRISPR/Cas9 technologies have revolutionized the ability to interrogate gene function with precision. In HB research, CRISPR-based approaches enable systematic dissection of developmental signaling pathways, identification of essential genes, and modeling of specific mutations observed in patient tumors. These tools allow researchers to move beyond correlative associations toward causal understanding of tumor biology [112,113].

Functional genomic screens may also uncover synthetic lethal interactions and novel therapeutic targets, particularly within stemness-associated and resistance-driving pathways. When combined with organoid or PDX systems, CRISPR approaches provide a powerful framework for translational discovery [114].

### 9.4. Artificial Intelligence and Multi-Omics Integration

The growing availability of genomic, transcriptomic, epigenomic, and spatial data has created unique opportunities and challenges for HB research. Artificial intelligence and machine-learning approaches offer the means to integrate these complex datasets, identify hidden patterns, and generate predictive models of treatment response and outcome [115].

Multi-omics integration has the potential to redefine risk stratification by capturing biological complexity that exceeds the capacity of single-parameter models [116]. In the long term, AI-driven analyses may support clinical decision-making by linking molecular profiles with therapeutic recommendations, thereby operationalizing precision medicine in pediatric HB [117].

Together, advanced experimental models and computational approaches are reshaping the translational landscape of HB. By enabling functional validation of molecular insights and supporting patient-specific therapeutic exploration, these tools provide the infrastructure necessary to translate biological understanding into clinical impact. Their integration into future clinical trials will be essential for moving beyond empiric therapy toward truly precision-guided care [118,119].

### 9.5. Data Integration, Standardization, and Limitations of Precision Medicine in HB

The increasing emphasis on precision medicine in HB reflects major advances in molecular profiling and translational oncology. However, the successful implementation of biomarker-driven strategies remains constrained by significant challenges related to data heterogeneity, methodological variability, and limited prospective validation. These limitations are particularly relevant in rare pediatric tumors such as HB, where data are inherently fragmented across institutions and study designs [28,29].

Clinical heterogeneity represents a first major barrier. Although systems such as PRETEXT have improved risk stratification and surgical planning, variations in imaging acquisition, interpretation, and reporting persist across centers. Differences in chemotherapy protocols, timing of surgery, and criteria for transplantation further complicate cross-study comparisons and limit the generalizability of outcome data [19,120]. Even within established cooperative frameworks, inconsistencies in annotation factors or response assessment may introduce variability that affects both clinical decision-making and research conclusions.

At the molecular level, variability is even more pronounced. Differences in tissue sampling (biopsy versus resection), sample preservation, sequencing platforms, and bioinformatic channels can substantially influence the detection and interpretation of genomic and transcriptomic alterations. In HB, where the mutational burden is relatively low and key pathways such as Wnt/β-catenin dominate, subtle molecular signals may be obscured by technical noise if data are not generated and analyzed under standardized conditions [7,121]. This challenge extends to emerging biomarkers such as circulating tumor DNA (ctDNA) and microRNAs, where pre-analytical variables and assay heterogeneity remain significant limitations.

A critical issue arising from these limitations is the so-called “validation gap”. Much of the current knowledge regarding molecular subtypes, tumor microenvironment features, and potential therapeutic targets in HB is derived from retrospective, observational, or correlational studies. While these approaches are valuable for hypothesis generation, they do not establish causality or clinical utility. The translation of such findings into practice requires prospective clinical trials incorporating molecular stratification and standardized endpoints, which remain scarce in this rare disease setting [29,122].

Importantly, data standardization is not only essential for translational research but also has direct implications for surgical management. Accurate assessment of tumor resectability depends on high-quality, standardized imaging and consistent application of PRETEXT/POSTTEXT criteria. Variability in imaging interpretation may lead to either overly aggressive resections or missed opportunities for curative surgery. Similarly, reliable estimation of FLR through volumetric analysis requires standardized methodologies to minimize the risk of postoperative liver failure [120].

Post-chemotherapy evaluation represents another critical interface between oncology and surgery. The degree of histological response, including tumor necrosis and residual viable tumor, is an important prognostic factor and may influence subsequent therapeutic decisions. However, without standardized protocols for tissue processing, sampling, and reporting, these parameters may be inconsistently assessed. Likewise, uniform definitions of surgical margins (R0, R1, R2) and their clinical interpretation are essential for meaningful comparison of outcomes across centers and studies [28].

Looking forward, the integration of molecular risk stratification into surgical decision-making represents a key goal of precision medicine. For example, tumors with high-risk biological features may warrant more aggressive strategies, including early consideration of liver transplantation, whereas low-risk tumors could potentially benefit from treatment de-escalation. However, such approaches remain largely hypothetical in the absence of validated biomarkers and harmonized data frameworks.

Given the rarity of HB, no single institution can generate sufficiently powered datasets to address these challenges independently. Therefore, international collaboration and standardization are essential. Collaborative groups such as SIOPEL, COG, and GPOH play a central role in harmonizing clinical protocols, but further efforts are needed to standardize molecular analyses, data annotation, and data sharing infrastructures. The development of integrated, high-quality, and interoperable datasets will be critical to reduce noise, improve reproducibility, and enable robust biomarker validation [29,122].

In this context, without coordinated efforts toward data standardization and prospective validation, there is a substantial risk that precision medicine in HB will remain largely theoretical. Addressing this challenge represents not only a methodological necessity but also a strategic priority for advancing personalized care in this rare pediatric malignancy.

### 9.6. Access to Innovative Therapies and Global Disparities in Pediatric Oncology

Despite advances in HB management, access to optimal care remains highly unequal across regions. In low- and middle-income countries, limitations in infrastructure, availability of chemotherapy, access to LT, and molecular diagnostics significantly impact outcomes [123]. These disparities are further amplified in rare pediatric cancers, where expertise and resources are concentrated in specialized centers. Recent studies highlight that improving access requires not only resource allocation but also international collaboration, standardized protocols, and shared data platforms [124,125]. In addition, delayed diagnosis and limited inclusion in international clinical trials further contribute to outcome disparities, restricting access to novel therapeutic strategies in these settings. Strengthening global networks and facilitating access to molecular diagnostics may represent key steps toward reducing outcome inequalities and enabling the implementation of precision medicine approaches worldwide.

## 10. Future Perspectives

The evolving understanding of HB biology offers a unique opportunity to redefine therapeutic paradigms in pediatric liver cancer. As survival rates continue to improve with conventional strategies, the focus increasingly shifts toward optimizing treatment intensity, minimizing long-term toxicity, and addressing biologically aggressive disease more effectively. Achieving these goals will require a fundamental transition from anatomy-centered decision-making to an integrated, biology-informed framework [18,115].

### 10.1. Toward Integrated Molecular and Surgical Stratification

A central future direction lies in the integration of molecular stratification with surgical planning. Current treatment algorithms prioritize anatomical resectability and radiologic staging, yet accumulating evidence suggests that molecular features, such as activation of developmental signaling pathways, stemness-associated programs, and epigenetic states, may critically influence tumor behavior, response to therapy, and risk of recurrence. Incorporating molecular information into preoperative assessment could refine decisions regarding upfront resection, neoadjuvant therapy intensity, and transplant candidacy [126].

Such an approach has relevance for borderline-resectable tumors, where biological aggressiveness rather than anatomy alone may determine optimal timing and extent of surgical intervention. Molecularly informed stratification could thus transform surgery from a purely technical endpoint into a biologically contextualized component of precision therapy [18].

### 10.2. Personalized Therapeutic Strategies

The future management of HB is likely to involve increasingly personalized therapeutic approaches tailored to specific biological vulnerabilities. Advances in molecular profiling, liquid biopsy, and functional modeling provide the tools necessary to move beyond empiric treatment escalation. Instead, therapy may be adapted to target dominant oncogenic pathways, disrupt stem-like tumor compartments, or modulate the tumor microenvironment in a patient-specific manner [2,51,127].

Importantly, personalization does not necessarily imply treatment intensification. For tumors exhibiting favorable molecular profiles and differentiated phenotypes, therapy de-escalation may be both safe and desirable, reducing exposure to cytotoxic agents and mitigating long-term sequelae. Conversely, biologically high-risk tumors may benefit from early incorporation of innovative strategies within carefully designed clinical trials [18].

### 10.3. Therapeutic De-Escalation as a Biological Strategy

Therapeutic de-escalation represents a critical yet underexplored frontier in HB care [128]. As molecular tools improve risk discrimination, it may become possible to identify subsets of patients for whom reduced chemotherapy intensity or shortened treatment duration does not compromise cure rates. Such strategies would align oncologic success with the long-term health and quality of life of survivors, a paramount consideration in pediatric oncology [18,129].

However, de-escalation must be guided by robust biological markers rather than clinical response alone. Integrating molecular, epigenetic, and functional data into prospective trials will be essential to validate safe de-escalation strategies and to avoid undertreatment of biologically aggressive disease [130].

**Figure 6:**
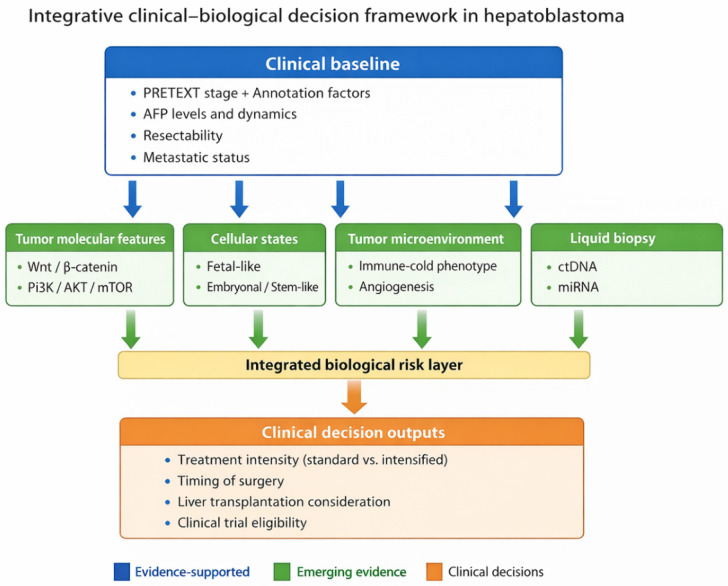
Integrative clinical–biological decision framework in hepatoblastoma. This framework illustrates how these complementary layers may converge into a unified biological risk profile, ultimately informing key clinical decisions such as treatment intensity, surgical strategy, transplantation, and trial enrollment.

To translate these concepts into a clinically relevant perspective, we propose an integrative decision framework that combines established clinical parameters with emerging biological and biomarker-driven dimensions (Figure 6).

While this model remains partially conceptual, it provides a structured approach for future validation and integration of multi-dimensional biomarkers into clinical practice.

## 11. Conclusions

Hepatoblastoma is increasingly recognized as a malignancy rooted in disrupted liver development rather than a conventional solid tumor driven by cumulative genetic damage. Its biology reflects arrested differentiation, aberrant activation of developmental signaling pathways, and the persistence of stem-like cellular states, all of which contribute to clinical heterogeneity and variable therapeutic response. HB management remains anchored in multimodal therapy combining chemotherapy and complete surgical resection. While advances in molecular profiling and tumor biology provide promising avenues for precision medicine, their clinical implementation is still limited by insufficient validation and data heterogeneity. Emerging therapeutic strategies should therefore be considered complementary and investigational rather than substitutive. Future progress will depend on the integration of standardized clinical and molecular data within international collaborative frameworks, enabling more robust and equitable implementation of personalized approaches.

## Figures and Tables

**Table 1:** Histopathological subtypes of hepatoblastoma and clinical correlates [30].

Histological Subtype	Key Features	Typical AFP	Clinical Behavior	Prognostic Implication
Well-differentiated fetal	Mature hepatocyte-like cells	High	Slow growth	Favorable
Embryonal	Primitive cells, high mitoses	High	Aggressive	Intermediate–poor
Mixed epithelial–mesenchymal	Stromal elements	Variable	Variable	Intermediate
Small cell undifferentiated	Sheets of small cells	Often low	Highly aggressive	Poor

**Table 2:** Major Molecular Pathways Implicated in HB Heterogeneity [2].

Pathway	Key Alterations	Biological Effect	Clinical Relevance
Wnt/β-catenin	CTNNB1 mutations	Developmental arrest	Core oncogenic driver
Hippo–YAP	YAP activation	Proliferation, survival	Aggressiveness
IGF	IGF2 overexpression	Growth, metabolism	Targetable axis
mTOR	Pathway activation	Chemoresistance	Therapeutic target
Notch	NOTCH activation	Stemness, fate control	Potential target

**Table 3:** Clinical Implications of Histological and Molecular Diversity.

Feature	Clinical Impact	Therapeutic Implication
Predominant fetal phenotype	Good response	Consider therapy de-escalation
Embryonal/stem-like profile	Poor response	Intensification/trials
High intratumoral heterogeneity	Relapse risk	Combination strategies

**Table 4:** Proposed biomarker-informed combination strategies in hepatoblastoma: biological rationale, therapeutic integration, and clinical considerations.

Therapeutic Axis	Biological Rationale	Potential Combination Partners	Candidate Biomarkers	Clinical Integration	Pediatric Considerations
mTOR pathway inhibition	Activated PI3K/AKT/mTOR signaling in proliferative and resistant tumor compartments	Cisplatin-based chemotherapy; anti-angiogenic agents	p-AKT, p-mTOR, gene expression signatures	High-risk or refractory disease; post-induction intensification	Growth/metabolic effects; long-term toxicity
Wnt/β-catenin targeting	Central driver in HB tumorigenesis and differentiation arrest	Epigenetic modulators; YAP/TAZ inhibition (hypothesis-driven)	Nuclear β-catenin, CTNNB1 mutations	Currently experimental; potential in refractory or relapsed settings	Limited pediatric safety data
Hippo–YAP/TAZ axis	Associated with stem-like states and tumor aggressiveness	Wnt inhibitors; cytotoxic chemotherapy	YAP nuclear localization, TEAD signatures	High-risk tumors with progenitor-like phenotype (hypothesis-generating)	Unknown long-term effects
Anti-angiogenic therapy	Vascular remodeling and tumor growth dependency	Chemotherapy; mTOR inhibitors	VEGF expression, microvessel density	Selected advanced/metastatic cases; perioperative context	Hypertension, growth impact
Immunotherapy	Immune-cold microenvironment but potential in selected niches	Anti-angiogenic agents; epigenetic therapy	PD-L1, immune infiltration signatures, ctDNA dynamics	Currently investigational; possible in relapsed disease	Immune-related toxicity considerations
Epigenetic modulation	Aberrant differentiation programs and transcriptional plasticity	Wnt/YAP targeting; chemotherapy	DNA methylation patterns, histone markers	Experimental; potential for differentiation therapy	Safety profile not fully defined

**Table 5:** Key studies/trials informing innovative or biomarker-enabled strategies in hepatoblastoma.

Strategy/Axis	Setting	Study/Trial	Design	Main Message Relevant to “Innovative Strategies”	Citation	Evidence Level/Boundary
Risk-adapted, biomarker-enabled international platform (framework for therapy + future molecular layering)	Newly diagnosed HB/HCC	PHITT	International umbrella/platform trial	Establishes a harmonized, risk-adapted backbone suitable for integrating biological stratifiers and future targeted components within a cooperative-group framework	[87]	Evidence-supported platform (infrastructure enabling prospective biomarker integration)
De-escalation/toxicity reduction within risk-based care	Very low/low-risk HB	AHEP0731 (COG)	Phase III multicenter trial	Demonstrates that risk-adapted reduction in therapy intensity is feasible in selected groups; provides a template for future biomarker-informed de-escalation	[88,89]	Evidence-supported (practice-informing for selected risk groups)
High-risk intensification backbone—reference standard of care (SOC) for combinations	High-risk HB	SIOPEL-4	Prospective single-arm feasibility study	Defines a cisplatin-intensive reference approach and feasibility constraints; useful SOC backbone when proposing add-on targeted/immune agents	[65]	Evidence-supported (SOC backbone; not “novel agent” proof)
Standard-risk chemotherapy backbone (benchmark comparator)	Standard-risk HB	SIOPEL-3 (cisplatin vs. cisplatin + doxorubicin)	Randomized trial	Establishes evidence-supported chemotherapy backbone; clarifies what is already validated vs. investigational add-ons	[64]	Evidence-supported (SOC comparator for add-on strategies)
Prospective molecular risk-predictive model embedded in therapy	Newly diagnosed pediatric hepatic malignancies including HB	AHEP1531/HB-MRP evaluation	Prospective platform with biomarker model evaluation	Provides a route for prospective validation of biomarker-driven risk prediction, addressing the “validation gap” via cooperative-group embedding	[90]	Emerging evidence/prospective validation (biomarker utility being tested)
Novel-agent “window” exploring non-platinum combinations (pilot feasibility)	High-risk HB	Vincristine/Irinotecan window within AHEP0731 HR stratum	Prospective exploratory “window” component	Illustrates how non-platinum regimens are piloted within cooperative protocols; informs feasibility and signal-seeking, not definitive efficacy	[91]	Early clinical/hypothesis-generating (signal-seeking)
Immune checkpoint-based combination (innovative medicine trial)	Advanced relapsed/refractory HB	Pucotenlimab + lenvatinib + chemotherapy	Interventional pediatric trial	Represents current generation IO + anti-angiogenic combinations entering pediatric HB trials; supports discussion of patient-selection and toxicity constraints	[92]	Investigational/early-phase (ongoing; efficacy not established)
mTOR pathway targeting (representative preclinical rationale)	Preclinical HB models	Rapamycin (mTOR inhibition)	Preclinical in vitro/in vivo	Pathophysiological basis for PI3K/AKT/mTOR targeting; should be framed as rationale until supported by stronger pediatric HB clinical evidence	[93]	Preclinical/hypothesis-generating

## Data Availability

No new data were created or analyzed in this study. Data sharing is not applicable to this article.

## Abbreviations

AFP: Alpha-fetoprotein; CHIC: Children’s Hepatic Tumors International Collaboration; CK: Cytokeratins; COG: Children’s Oncology Group; CRISPR: Clustered regularly interspaced short palindromic repeats; CTNNB1: Catenin Beta 1; ctDNA: Circulating tumor DNA; EpCAM: Epithelial cell adhesion molecule; FLR: Future liver remnant; GBD: Global Burden of Disease; GPOH: German Society of Pediatric Oncology and Hematology; HB: Hepatoblastoma; HE: Hematoxylin and eosin; IGF: Insulin-like growth factor; LT: Liver transplantation; miRNAs: MicroRNAs; PDX: Patient-derived xenografts; POSTTEXT: Post-treatment extent of disease; PRETEXT: Pretreatment extent of disease; SIOPEL: International Society of Paediatric Oncology–Epithelial Liver Tumour Group; SOC: Standard of care; VEGF: Vascular endothelial growth factor.
